# Recent trends in energy and nutrient content of take-home food and beverage purchases in Great Britain: an analysis of 225 million food and beverage purchases over 6 years

**DOI:** 10.1136/bmjnph-2019-000036

**Published:** 2019-08-01

**Authors:** Nicolas Berger, Steven Cummins, Richard D Smith, Laura Cornelsen

**Affiliations:** 1 Population Health Innovation Lab, Department of Public Health, Environments and Society, London School of Hygiene and Tropical Medicine, London, UK; 2 College of Medicine and Health, University of Exeter, Exeter, UK

**Keywords:** calories purchased, diet, nutrients purchased, scanner data, trends, united kingdom

## Abstract

**Introduction:**

In recent years, there has been an increased focus on developing a coherent obesity policy in the UK, which has led to various national policy initiatives aimed at improving population diet. We sought to determine whether there have been concurrent changes in trends in the nutrient content of take-home food and beverage purchases within this policy environment.

**Methods:**

We used 2012–2017 data from the UK Kantar Fast-Moving Consumer Goods (FMCG) panel, a nationally representative panel study of food and beverages bought by British households and brought into the home (n≈32 000 per year). Households used hand-held barcode scanners to report over 225 million product-level purchases of food and beverages, for which nutritional information was obtained. We estimated daily per capita purchases of energy and nutrients from 32 healthier and less healthy food groups defined using the nutrient profiling model used by the UK Department of Health.

**Results:**

From 2012 to 2017, daily purchases of energy from food and beverages taken home decreased by 35.4 kcal (95% CI 25.5 to 45.2) per capita. This is explained by moderate decreases in the purchase of products with high contents in carbohydrate (−13.1 g (−14.4 to –11.8)) and sugar (−4.4 g (−5.1 to –3.7)), despite small increases in protein (1.7 g (1.4 to 2.1)) and saturated fat (0.4 g (0.2 to 0.6)). Food and beverage purchases exceeded daily reference intake values in fat (on average +6%), saturated fat (+43%), sugar (+16%) and protein (+28%) across all years. Although substitutions between individual food groups were large in energy and nutrients purchased, the heterogeneity of these patterns resulted in modest overall changes.

**Conclusion:**

There have been small declines in the purchase of less healthy food products, which translated to a small reduction of total energy and sugar purchases taken home. However, the rate of change needs to be accelerated in order to substantially reduce the health risks of poor diets, suggesting that more radical policies may be needed to attain larger population effects.

**What this paper adds**

- Food and beverages that people buy and take home exceed recommended levels for fat, saturated fat, sugar and protein intake.
- From 2012 to 2017 the energy content of these take-home purchases declined a little (by 35.4 kcal per person per day), which is primarily explained by a decrease in the purchase of products with high sugar and carbohydrate content, despite protein and saturated fat content increasing at the same time.
- The rate of change in food and beverage purchase habits needs to be accelerated to substantially reduce the health risks of poor diets.

## Introduction

Despite small improvements since the late 1980s,^1^ the majority of the UK population fall short of meeting dietary recommendations; consuming too much salt, added sugar and saturated fat, and too few fruits, vegetables, fibre and oily fish.^2^ With more than a quarter of adults and a fifth of 10-year-old children obese, there is now a major concern about dietary risks as a national public health problem.^3–5^

Recent years have seen a greater focus on developing a coherent obesity policy in the UK.^6–9^ This has led to a raft of national policy initiatives aimed at improving diet and reducing obesity. As early as 2004, the UK government set the target of reducing the average salt intake of adults to 6 g/day through a voluntary industry reformulation programme of selected products high in salt content (2006, 2009). Subsequent salt reduction targets were integrated in the Public Health Responsibility Deal (2011–2015), which also included a series of public–private food pledges aiming to reduce calorie, salt and saturated fat intake, provide food labelling and increase fruit and vegetable consumption to reach the ‘5 a day’ targets launched in 2003.^10^ In 2016, a Soft Drinks Industry Levy was announced to be implemented in 2018, providing time for the industry to reformulate targeted drinks. The Childhood Obesity Strategy was also launched in 2016, which further included a voluntary Sugar Reduction Programme for the industry targeting food products high in sugar content.^11^ This period was also characterised by factors, which could have reinforced the national policy actions, these include the publication of WHO guidelines on the intake of sugar (2015)^12^ and processed and red meat (2016),^13^ as well as extensive media coverage on dietary risks, obesity and related policies (eg, see ref. 14, for an analysis of the media coverage of the health risks related to sugar-sweetened beverages).

While it is too early to tell if these initiatives will reduce obesity or disease prevalence, it is reasonable to expect that, if these were successful, we would start seeing some positive changes in household food and energy purchase behaviour. In particular, given the focus of the policies on salt and sugar reductions, a decline in the overall purchases of these nutrients could be expected. Previous studies, focused on the early stage of the salt reduction programme, have found a gradual decrease towards the recommended lower level of salt intake.^15–17^ However, to our knowledge, no study has explored recent food purchase trends covering the whole basket of foods across different nutrients. This information is critical for ongoing policy initiatives through food industry voluntary actions as well as legislative or any other type of actions and public health interventions.

In this paper, we investigate trends in the amount of energy and nutrients bought by British households to take-home between 2012 and 2017. Considering the policy context, we analyse what types of foods or beverages are key contributors to energy and nutrients purchased and whether there have been changes towards purchasing healthier products in recent years.

## Methods

### Study population

Data were obtained from the UK Kantar FMCG panel, a consumer panel of food and beverages purchased by households in Great Britain (GB) and brought into their home. Our dataset covers purchases during 2012–2017. The panel uses an open-panel design, comprising 31 000–34 000 households annually. Households are recruited via stratified sampling, with quotas set for region, household size, age of main shopper, number of children and occupation. Households record purchases continuously throughout the year and are offered incentives to remain in the panel in the form of vouchers with an average value of £100 per household per year. Panel retention is high—participating households in 2012 had mean follow-up time of 4.1 years. Approximately, 3000–4000 new households are enrolled each year to maintain national representativeness. Panellists provide sociodemographic data when joining the panel (including, age, sex, occupation, ethnicity, household composition, income and others) followed by annual updates. The dataset further includes gross-up weights to derive population-level estimates by accounting for the sampling design and non-response.

### Food and beverage purchase data

Households record food and beverage purchases brought back into the home using hand-held barcode scanners. Places of purchase include supermarkets, convenience stores, newsagents and specialist stores such as butchers, greengrocers. Non-barcoded products, such as loose fruits and vegetables, are recorded using bespoke barcodes. Participants additionally provide price information from receipts. Kantar FMCG collects nutritional data on products purchased through direct measurement in outlets twice a year, or using product images provided by Brandbank, a third party supplier. Where Kantar is unable to gather direct information, nutritional values are either copied across from similar products or an average value for the category or product type is calculated and used instead (the proportion of imputed values was lowest for energy (11.0%) and highest for fibre (19.6%)). Data available for analyses included n=225 036 065 item-level observations of food and beverage product purchases over six 52-week periods. Outcomes used in this study are: energy (kcal), fat (g), protein (g), carbohydrate (g), sugar (g), saturated fat (g), sodium (g) and non-starch polysaccharides (NSP) fibre (g).

### Data cleaning and exclusion criteria

We identified potential incorrect values using logic checks and summary statistics. Overall, we corrected one variable or more in 1.6% of all transactions, after consultation with Kantar. The majority of corrections related to nutritional information, but for a small number of observations measurement units and pack numbers were also modified to ensure consistency. Finally, we investigated the products with corrected transactions, and excluded products with inconsistent time series (5% of observations) to ensure quality of the data analysed. We estimated that these products could account for up 130 kcal/capita/day. The final sample included n=213 663 901 item-level observations (see online supplementary figure 1).

10.1136/bmjnph-2019-000036.supp1 Supplementary data

### Food group classification

We grouped products into 32 distinct categories based on a previously used food group classification (table 1)^18 19^ that separates products into predefined categories (eg, bread, cheese and savoury snacks). We further separated healthier from less healthy products within each category using the UK Department of Health nutrient profiling model, a model widely used for policy actions and supported by the UK Scientific Advisory Committee on Nutrition.^20^ The nutrient profiling model assigns points to products based on the content of energy, sugar, saturated fat, salt, NSP fibre, protein and fruit and vegetables. Food products scoring above 4 points and drinks scoring above 1 point are classified as less healthy (see ref. 20 for details of score calculation). For some categories such as cheese, fruits and vegetables, the differentiation was not necessary due to more than 90% of products falling into either healthier or less healthy categories. Table salt, alcoholic beverages and juices were not scored and were all classified as less healthy.^21^ The final classification included 14 healthier food groups and 18 less healthy food groups (table 1).

**Table 1:** Food group classification and nutrient profiling score

	Nutrient profiling score Mean±SD	
	Healthier*	Less healthy
Paired food groups		
Bread products†	−2.00±2.89	13.12±5.02
Breakfast cereals	−3.08±2.53	8.28±2.69
Other dairy (incl. cream, yoghurt, fromage frais)	−0.07±2.06	9.99±4.81
Red meat	−0.09±2.34	9.01±4.28
Processed fish and meat†	0.59±2.03	12.39±5.76
Ready meals and convenience food	−0.40±2.29	10.67±3.90
Sauces and condiments	0.00±2.64	11.85±5.56
Other drinks (incl. water, cordials, carbonated, dairy based)	−0.32±1.52	6.69±8.54
Healthier food groups		
Pasta, rice, potatoes and other grains	−4.19±4.35	
Proteins (incl. egg, fish, white meat, meat substitutes)	−0.25±3.81	
Fruits	−4.01±2.02	
Vegetables, excl. legumes and potatoes	−6.71±3.20	
Legumes, nuts and seeds	−8.75±4.34	
Milk	0.06±1.53	
Less healthy food groups		
Other morning goods (incl. croissants, pastry, crumpet)†		14.97±8.80
Savoury snacks		10.91±5.41
Chocolates and confectionery†		19.72±7.17
Puddings and biscuits†		14.58±7.36
Cheese		18.79±5.62
Fat and oil		20.92±3.45
Caloric sweeteners (sugar, honey and syrup)		13.96±1.16
Table salt‡		–
Juices		–
Alcohol		–

*Food products scoring above 4 points and drinks scoring above 1 point are classified as less healthy. Food groups were slit into pairs if at least 10% of products were classified as healthy/less healthy. Otherwise, products are assigned to a healthier/less healthy food group.

†Some products were excluded due to unreliable nutritional data.

‡Only sodium information is reported for table salt.

### Statistical analysis

To capture population-level changes in take-home purchases, we estimated average daily energy and nutrient purchases per capita for each year between 2012 and 2017, overall and by food group. Estimates were derived in two steps: first, we applied gross-up weights to estimate the total energy and nutrient purchases in a given year for the whole GB population (ie, total market); second, we calculated daily purchases per capita by dividing the total energy and nutrient purchases by annual population size (provided by Kantar FMCG) and by the number of days in a given year. Annual-level estimates were chosen to best capture shopping habits throughout the year and to account for storage of products not consumed immediately.^22^ Cluster robust SEs were used to account for clustering at household level.^23^

Total estimates were compared against reference intake (RI) values for adults set by European legislation, and national recommendations for fibre.^24 25^ Finally, linear time trends were tested with random-effects meta-regression analyses using time as a predictor and the inverse of the annual variances as weights. Data analyses were performed using Stata MP V.15.1.^26^

## Results

We analysed n=213 663 901 products purchased from n=50 672 households. The number of active households per year was highest in 2014 (n=33 309) and lowest in 2017 (n=31 725). Table 2 summarises household sociodemographic characteristics.

**Table 2:** Household characteristics: UK Kantar FMCG panel 2012–2017 (N=50 672)

Variable	Category	Summary statistic*
Age of main shopper (years)		49.9±15.2
Occupation group	A and B: higher managerial and professional workers	21.2
	C1 and C2: white collar and skilled manual workers	56.0
	D and E: semiskilled and unskilled manual workers	22.8
Ethnicity of main shopper†	Non-White	6.5
No of household members	1	18.1
	2	34.6
	3	19.2
	4+	28.1
No of children in household	0	64.2
	1	15.9
	2	14.7
	3	4.0
	4+	1.2
Region	London	16.1
	Midlands	15.1
	North East	4.9
	Yorkshire	12.8
	Lancashire	10.8
	South	10.7
	Scotland	8.9
	Anglia	8.7
	Wales and West	8.6
	South West	3.4
Total households by year (N)‡	2012	32 726
	2013	32 620
	2014	33 309
	2015	32 887
	2016	32 110
	2017	31 725

*Values are percentages for categorical variables and mean±SD for continuous variables.

†Ethnicity is missing for 5.7% of households.

‡The total number of households is smaller than the sum of households of each year because most households stayed in the panel for longer than a year.

### Trends in energy and nutrient content of purchases

**Figure 1:**
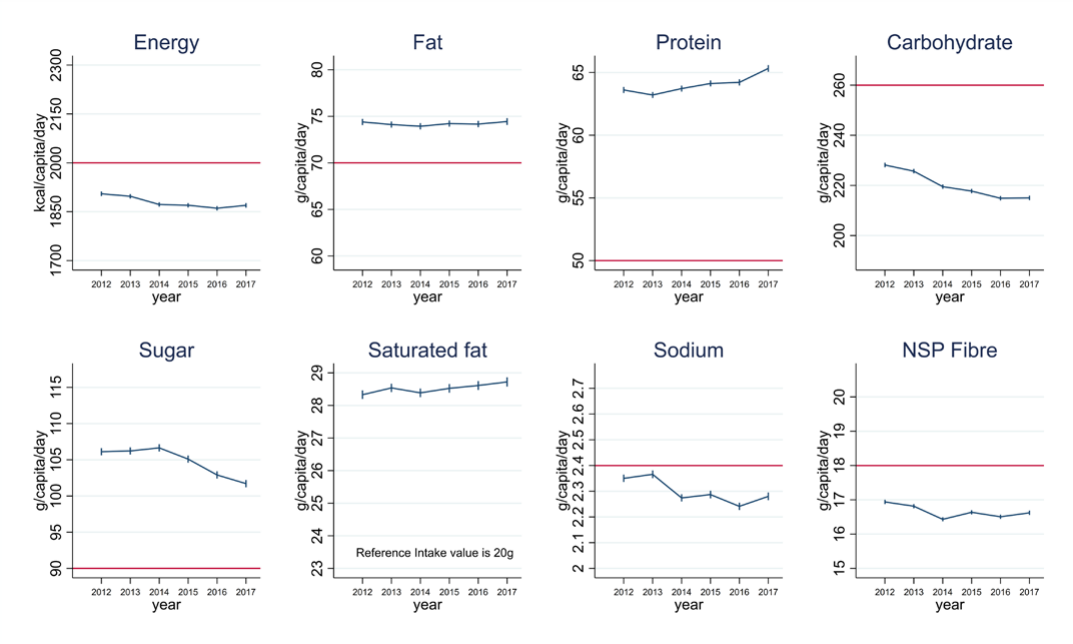
Daily energy and nutrient purchases per capita in Great Britain. Data are from 213 663 901 transactions reported by 50 672 households participating in UK Kantar FMCG panel between 2012 and 2017. Values are mean daily per capita energy and nutrient purchased to take-home. The red horizontal line indicates the reference intake values and the national recommendation of 18 g for NSP fibre, which is equivalent to 30 g of fibre. The ranges of the y-axes are 30% of the reference intake values so that changes in the y-axes and differences with the reference intake values are visually comparable across nutrients. When the differences with the reference intake values were within 15%, the y-axes were centred on the reference intake value. The difference between saturated fat purchases and the reference intake value was greater than 30% so that it could not be displayed on the graph. Some puddings, biscuits and bread products, as well as all bacon and sausages, slimming products and milkshake mixes were excluded because of inconsistent nutrient information reported at product level. Products excluded could account for up 130 kcal/capita/day each year. NSP, non-starch polysaccharides.

The average energy content of daily food and beverage purchases taken home decreased by 35.4 kcal per capita (95% CI 25.5 to 45.2; p-trend=0.023) between 2012 and 2017 (figure 1, online supplementary table 1). Fat, saturated fat, protein and sugar content of purchases were all above the RI values throughout the study period. In particular, saturated fat and protein contents were more than 25% above RI limits. Conversely, purchases of carbohydrate, sodium and fibre were below the RI values. While the fat content of daily purchases stayed around 74 g per capita (p-trend=0.757), the saturated fat content increased marginally from 28.3 to 28.7 g (p-trend=0.031), and protein content increased from 63.6 g (63.4 to 63.9) to 65.3 g (65.1 to 65.6; p-trend=0.022).

Sugar content of purchases was stable between 2012 and 2014, followed by a 4.9 g (4.2, 5.6) decrease between 2014 and 2017 (from 106.6 to 101.7 g, overall p-trend=0.018). Carbohydrate content of purchases decreased by 13.1 g (11.8 to 14.4) between 2012 and 2017 (from 228.1 to 215.0 g; p-trend=0.003) (of which 4.4 g were the above mentioned sugar reduction). We also note small decreases in content of sodium and fibre purchases from 2.35 g (2.34 to 2.36) to 2.28 g (2.26 to 2.30; p-trend=0.061), and 16.9 g (16.9 to 17.0) to 16.6 g (16.6 to 16.7; p-trend=0.160), respectively.

### Food group contributions to total energy purchased

In 2012, 45.0% of the total energy was purchased from healthier food groups (table 3). This increased to 46.1% by 2017. Biggest contributors among the healthier foods were bread products (176.4 kcal/capita/day (175.4 to 177.4) in 2012), healthier ready meals and convenience food (107.5 kcal (106.9 to 108.1)), pasta, rice, potatoes and other grains (126.6 kcal (125.8 to 127.5)) and milk (120.3 kcal (119.2 to 121.3)).

**Table 3:** Changes in food group contributions to energy and nutrient purchased (UK Kantar FMCG panel 2012–2017)

	Δ 2017−20121*								
	Kcal 2012	Energy (kcal)	Fat (g)	Protein (g)	Sugar (g)	Carb.† (g)	Sat. fat (g)	Sodium (g)	Fibre (g)
Healthier food groups									
Pasta, rice, potatoes and other grains	126.6‡	−18.1	−0.2	−0.5	−0.2	−3.3	0.0	−0.01	−0.11
Bread products§	176.4	−13.3	−0.1	−0.3	−0.1	−2.5	0.0	−0.03	−0.26
Fruits	65.2	−1.1	0.1	0.1	0.9	−1.6	0.1	0.00	0.06
Milk	120.3	−0.2	−0.3	0.2	0.3	0.0	−0.1	0.01	−0.05
Other dairy§	20	0.0	0.0	0.1	−0.1	−0.1	0.0	0.00	−0.02
Legumes, nuts and seeds	26.6	0.3	0.2	−0.1	−0.1	−0.3	0.0	−0.01	−0.09
Sauces and condiments§	2.4	1.1	0.1	0.0	0.0	0.0	0.0	0.00	0.02
Other drinks§	8.4	2.1	0.1	0.0	0.2	0.1	0.0	0.01	−0.02
Ready meals and convenience food§	107.5	2.6	0.1	0.2	0.0	−0.2	0.1	0.00	−0.02
Proteins	75.6	3.9	0.2	0.8	0.0	0.0	0.0	0.01	0.02
Red meat§	23.3	5.1	0.2	0.5	0.0	0.0	0.1	0.00	0.00
Processed fish and meat§	24.8	5.3	0.2	0.4	0.0	0.2	0.0	0.01	0.00
Breakfast cereals§	37.9	8.0	0.1	0.2	0.2	1.2	0.0	0.00	0.12
Vegetables, excl. legumes and potatoes	42	10.0	0.2	0.2	0.6	0.7	0.0	0.01	0.28
**Subtotal**	**857.0**	**5.7**	**0.83**	**1.78**	**1.58**	−**5.92**	**0.21**	**0.01**	−**0.10**
Less healthy food groups									
Fat and oil	162.9	−15.1	−1.6	0.0	0.0	0.0	−0.2	−0.03	−0.01
Caloric sweeteners (sugar, honey, syrup)	58.4	−11.4	0.0	0.0	−2.6	−0.2	0.0	0.00	0.00
Breakfast cereals§	44.8	−9.5	0.0	−0.2	−0.7	−1.4	0.0	−0.02	−0.10
Red meat§	24.3	−7.8	−0.6	−0.7	0.0	0.0	−0.3	−0.01	0.00
Puddings and biscuits	184.1	−6.8	−0.1	−0.2	−0.5	−1.0	0.1	0.00	−0.08
Juices	29.2	−6.1	0.0	−0.1	−1.4	0.0	0.0	−0.01	−0.02
Ready meals and convenience food§	57.9	−6.0	−0.4	−0.1	0.0	−0.6	−0.1	−0.01	−0.08
Other drinks§	28.6	−4.8	0.0	0.0	−0.9	−0.3	0.0	−0.01	0.00
Alcohol	74.7	0.4	0.0	0.0	0.0	0.4	0.0	0.00	0.00
Other morning goods	25.4	0.7	0.0	0.0	0.1	0.1	0.0	0.00	0.01
Other dairy§	24.6	0.8	0.2	0.0	−0.2	0.0	0.1	0.00	−0.01
Bread product§	28.1	1.5	0.0	0.0	0.0	0.2	0.0	0.00	0.01
Sauces and condiments§	28.2	1.9	0.2	0.0	0.0	−0.1	0.0	0.00	0.02
Chocolates and confectionery	105.3	3.8	0.4	0.1	0.2	−0.5	0.2	0.00	0.02
Processed fish and meat§	36.5	3.9	0.2	0.6	0.0	−0.1	0.1	0.02	0.00
Cheese	59.2	6.0	0.5	0.4	−0.1	0.1	0.3	0.01	−0.02
Savoury snacks	75.6	7.4	0.4	0.1	0.1	0.6	0.0	0.01	0.06
Table salt	–	–	–	–	–	–	–	−0.03	–
Subtotal	1047.8	−41.0	−0.78	−0.07	−5.98	−2.79	0.18	−0.08	−0.22
Tota**l**¶	1904.9	−35.4	0.04	1.72	−4.40	−8.72	0.39	−0.07	−0.32

*Energy and nutrients changes given as /capita/day. The colour scales highlight the intensity of changes using distribution quintiles.

†Carbohydrate excludes sugar.

‡The full results with 95% CI are available in online supplementary tables 2 and 3.

§Paired food group (healthier vs less healthy).

¶Some puddings, biscuits and bread products, as well as all bacon and sausages, slimming products and milkshake mixes were excluded because of inconsistent nutrient information reported at product level. Products excluded could account for up 130 kcal/capita/day each year.

Carb, carbohydrate; Sat. fat, saturated fat.

Among the less healthy foods, the biggest contributors were puddings and biscuits (184.1 kcal (183.3 to 184.9) in 2012), fat and oil (162.9 kcal (161.8 to 164.0)), chocolates and confectionery (105.3 kcal (104.6 to 105.9)) and savoury snacks (75.6 kcal (75.1 to 76.0). In comparison to other less healthy food groups, juices and other less healthy drinks contributed considerably less (57.9 kcal combined).

### Change in food groups contributions to total energy

Table 3 also presents changes in energy and nutrients obtained from each food group between 2012 and 2017. Significant changes in energy content are observed for 27 of the 32 food groups (online supplementary tables 2 and 3). Changes were very heterogeneous, varying from −18.1 kcal (−19.3 to –16.9) to +10 kcal (9.6 to 10.3) per capita per day. Overall, we observed a slight increase in energy purchased from healthier products (+5.7 kcal/capita/day), in particular from vegetables, and healthier breakfast cereals. Concurrently, we note a decline in energy purchased from less healthy products (−41.0 kcal), in particular from fat and oil, caloric sweeteners, and less healthy breakfast cereals. Despite these positive trends, relatively large daily decreases in energy purchased are also observed for healthier food groups such as pasta, rice, potatoes and other grains and healthier bread products. In addition, the purchase of energy from cheese and savoury snacks increased.

Analysis of the paired healthier/less healthy food groups indicates clear evidence of a trend towards healthier options in three out of the eight pairs (ie, breakfast cereals, ready meals and convenience food, and other drinks). For example, the daily energy obtained per capita from healthier breakfast cereals increased by 8.0 kcal (7.6 to 8.4) while it decreased by 9.5 kcal (−9.8 to –9.2) for less healthy options. As a result, healthier products provided the majority of energy from breakfast cereals in 2017 (45.5 kcal vs 35 kcal), which was not observed in 2012. A less encouraging pattern is seen for breads, with a slight increase in the purchase of energy from less healthy products (+1.5 kcal (1.2 to 1.7)) combined with a substantial decrease in healthier product purchases (−13.3 kcal (−14.7 to –12.0)).

### Change in food groups contributions across nutrients

Changes in nutrients obtained from each food group provide a finer-grained understanding of how the average diet might be shifting in GB. The overall trend in increasing protein content in 2012–2017 (+1.7 g/capita/day; +2.7%) is largely explained by increased purchases from healthier foods (+1.8 g/capita/day) (table 3, see also figure 1 and online supplementary table 1). The overall decrease in sugar content (by −4.4 g; −4.1%) is a combination of increased purchases from healthier foods (+1.6 g) and decreasing purchases from less healthy foods (−6.0 g). However, the decrease in carbohydrate excluding sugar (−8.7 g; −7.1%) is a combination of reduction from both healthier (−5.9 g) and less healthy food groups (−2.8 g).

#### Protein

The increase in protein content of purchases (+1.7 g) is driven by a few food groups, including ‘proteins’ (ie, egg, fish, white meat and meat substitutes), healthier red meat, processed fish and meat (from both healthier and less healthy sources) and cheese. The decrease observed in less healthy red meats, and pasta, rice, potatoes and other grains was insufficient to counterbalance those increases.

#### Sugar

Increased vegetable purchases and substitutions within fruit group towards higher sugar content fruits (eg, grapes), increased the quantity of sugar from healthier food groups. This was counterbalanced by reductions in purchases from less healthy products, in particular: caloric sweeteners (including table sugar), juices, other drinks and less healthy breakfast cereals.

#### Carbohydrate (excluding sugar)

Decreases in carbohydrate (−8.7 g) content of daily purchases are driven by decreases in the purchase of the main source of carbohydrate (pasta, rice, potatoes and other grains, and healthier bread products). Substitutions within the fruit group also led to changes in the quantity of non-sugar carbohydrate content, as well as the decrease in dessert and puddings.

#### Saturated fat

Although trends for fat and saturated fat are most likely to be affected by the exclusion of products such as desserts, bacon and sausages, we observed slight increases in purchases of saturated fat in both healthier and less healthy food groups. Despite a general decrease in energy and fat purchased from less healthy food groups, saturated fat content of purchases increased, which is mainly attributable to increases in purchases of cheese, chocolate and confectionery. The increase in saturated fat content of purchases from healthier food groups is mainly driven by red meat, and ready meals and convenience food. Noteworthy decrease in purchases of saturated fat from fat and oil products is also observed.

#### Other nutrients with fewer changes

Fat, sodium and fibre purchases displayed less clear time trends and changed modestly between 2012 and 2017 (figure 1). Heterogeneous changes across food groups were nonetheless observed for these nutrients so that some within food group changes were greater than the total changes (table 3). For the most part, the changes observed followed changes in energy purchased. Fat purchased mainly increased from fat and oil, and mainly decreased from cheese and savoury snacks; sodium mainly increased from bread products and fat and oil, and decreased from less healthy processed fish and meat, and cheese. Fibre content mainly decreased from bread products and increased from vegetables.

## Discussion

Considering the backdrop of increased public health policy activity in the UK, we looked at changes in energy and nutrient contents of take-home food and beverage purchases from 2012 to 2017, hypothesising that small positive changes could be expected. We found that the amount of energy from purchased food and beverages and taken home slightly decreased by 35.4 kcal per capita per day (−1.9%) in GB. This is primarily explained by decreases in the purchase of products with high sugar and carbohydrate contents (−4.1% and −5.8%, respectively), despite increases in protein and saturated fat (2.7% and 1.4%, respectively). Throughout the period, food and beverage purchases continued to exceed daily RI values of fat (on average +6%), saturated fat (+43%), sugar (+16%) and protein (+28%). As we focus on take-home purchases only, overall purchases are likely to be even more above the recommended level if out-of-home purchases, which account for 25-39% of household total food expenditures, are also considered.^27^

Some substitutions towards the healthier food groups were seen, with increases in energy from vegetables (+10.0 kcal per capita/day) combined with a decrease in oil and fat (−15.1 kcal), and caloric sweeteners (−11.4 kcal). Nevertheless, a continuous increase in purchased energy in some less healthy products such as savoury snacks and cheese is also observed (+7.4 kcal and +6.0 kcal per capita/day, respectively). Overall, although the extent of within-food group changes in energy and nutrients was large, the heterogeneity of trends between food groups resulted in modest overall change.

Our findings are consistent with previous reports that the majority of the UK population fall short of meeting dietary recommendations.^2^ The main trends were consistent with the latest figures available from nutrition survey data, documenting increased vegetable and meat consumption, and reduced consumption of potatoes, bread, fat and caloric sweeteners.^28^

The consistency of our findings with observed trends from nutrition surveys supports that these trends are likely to reflect true changes in energy purchased as opposed to substitution towards energy obtained from out-of-home purchases, given the stability of food waste in recent years^29^ and stable share of take-home and out-of-home expenditures during our study period. Family food statistics estimate out-of-home expenditures to account for about 27% of total food expenditure since 2002 (annual variation: ±1%).^30^

With respect to specific nutrients, we noted three prominent findings relevant to recent policy actions on diet and obesity: (1) a declining carbohydrate and increasing protein content of purchases, (2) a general reduction in sugar content of purchases and (3) an absence of linear reduction in sodium content of food purchases. The first of these trends could reflect the increasing popularity of low-carbohydrate and/or high-protein diets as means to lose weight^31^ despite current purchases already remaining below the RI level. Products providing high-levels of carbohydrate are also an important source of fibre,^32^ so current trends of reduced purchases of pasta, rice, potatoes and other grains, and bread products are likely to further exacerbate risk factors associated with low fibre consumption.

Most of the increase in protein appears to be animal based, a type of protein associated with premature mortality.^33^ Whereas we observed a stable level of protein obtained from red meat, we did find an increase in the purchase of processed fish and meat (both from healthier and less healthy products), which are known cancer risk factors, a finding that goes against WHO recommendations of reducing processed meat consumption.^13^

Reducing sugar consumption has been a strong policy focus since 2015.^12 24^ Excess sugar consumption, which is considered as a particular concern for children, led to introduction of media campaigns to make ‘Sugar Swaps’,^34^ a Soft Drinks Industry Levy, a voluntary reformulation programme for the industry and other initiatives of the Childhood Obesity Strategy.^11^ While the purpose of the paper is not to evaluate the effect of each of these initiatives, the trends observed do suggest that the concurrent, continuous drop in sugar purchases could, at least partly, be attributable to these efforts. The main sources of sugar from which we observe a decline as of 2015 were caloric sweeteners (including table sugar) (−18%), juices (−23%) and other less healthy drinks (−15%). One important source of energy and sugar where no change was observed was chocolate and confectionery. Reformulation of those products, therefore, appears to be a particularly important policy to further reduce sugar and energy consumption.

The UK is recognised for its leading role in policies targeting salt consumption.^35^ During the last decade, policies have resulted in a successful decrease in salt intake,^17^ so that the average adult now consume 8 g of salt per day.^36^ Our findings, however, indicate that sodium content of foods and beverages brought home is stagnant around the RI value. Considering this is only a take-home consumption perspective, it is unsurprising that overall consumption of sodium is still above recommendations. Increases in purchases of certain food groups, such as cheese and processed fish and meat, as well as reduction in table salt purchases, seem to indicate that changes in purchase behaviours—and not only reformulation—may explain current trends in salt purchases.

The main limitation of our analysis is, in common with other studies, that we were unable to account for out-of-home purchases, which account for 25-39% of total food expenditures. While expenditure share between take-home and out-of-home has been constant during the study period and inflation-adjusted expenditure in take-home purchases did not decrease, it remains possible that the composition of out-of-home food purchased, which tends to be energy-rich and incompatible with dietary guidelines,^37–39^ might also have changed. However, the lack of comparable data on the nutritional content of out-of-home purchases at this level of detail inhibits further analyses of this potential issue.

There are also potential limitations related to the type of data used as participants might suffer from fatigue bias, and with reporting becoming less accurate over time. Kantar monitors these potential biases by identifying and excluding problematic panellists. They further calculate gross-up weights to account for under-reporting of some specific products to ensure that the total population (market) estimates are as accurate as possible. Previous studies showed that UK Kantar FMCG panel data followed the patterns and trends seen in other data sources.^40 41^

We should also acknowledge the limitations of the dichotomous classification of products as either ‘healthier’ or ‘less healthy’ in the nutrient profiling model of the UK Department of Health. Recent studies have indicated that the consumption of a greater proportion of ‘less healthy’ products was not consistently associated with worse health outcomes in later life and further research on the predictive validity of the classification is warranted.^42–44^ Regardless, we chose to use this model because of its application in existing and proposed policy in the UK.^45 46^

Finally, while the aim of this paper was to investigate population-level changes in dietary behaviours, previous studies have indicated persistent socioeconomic inequalities in diets in the years preceding our study period.^1 47^ Future work is, therefore, needed to establish if the overall trends described in this paper might eclipse underlying heterogeneous trajectories across socioeconomic groups.

## Conclusion

In this study of a large nationally representative panel of British households, we detected subtle declines in the purchase of less healthy food products between 2012 and 2017, translating in a reduction of total energy and sugar content of purchases taken home. Though these improvements are relatively small, they are offset by different trends in protein, carbohydrate and saturated fat purchases, and could be further counterbalanced by changes in out-of-home purchases. Considering recent policy efforts, it is encouraging to see positive change on energy and sugar purchases, but, this rate of change needs to accelerate to meaningfully reduce the health risks associated with poor diet.
