# Supplementary material for: Recent trends in energy and nutrient content of take-home food and beverage purchases in Great Britain: an analysis of 225 million food and beverage purchases over 6 years
Source: BMJ Nutr Prev Health. 2019 Aug 1;2(2):63–71. doi: 10.1136/bmjnph-2019-000036 (PMC7664498; doi:10.1136/bmjnph-2019-000036)
Supplement: Supplementary data [file bmjnph-2019-000036supp001.pdf]

Online Supplementary materials

Supplementary Table S1 - Daily take-home energy and nutrient purchases per capita in Great Britain[1]

	2012		2013		2014		2015		2016		2017	
	Total	(95% CI)	Total	(95% CI)	Total	(95% CI)	Total	(95% CI)	Total	(95% CI)	Total	(95% CI)
Energy	1904.9	(1898.0, 1911.8)	1897.4	(1890.7, 1904.1)	1872.1	(1865.4, 1878.8)	1869.8	(1863.1, 1876.5)	1860.7	(1853.9, 1867.4)	1869.6	(1862.5, 1876.6)
Fat	74.4	(74.1, 74.7)	74.1	(73.8, 74.4)	73.9	(73.6, 74.3)	74.2	(73.9, 74.6)	74.2	(73.8, 74.5)	74.4	(74.1, 74.8)
Protein	63.6	(63.4, 63.9)	63.2	(63.0, 63.4)	63.7	(63.5, 64.0)	64.1	(63.9, 64.4)	64.2	(64.0, 64.5)	65.3	(65.1, 65.6)
Carbohydrate	228.1	(227.2, 229.0)	225.7	(224.8, 226.6)	219.5	(218.6, 220.4)	217.7	(216.9, 218.6)	214.9	(214.0, 215.7)	215.0	(214.1, 215.9)
Sugar	106.1	(105.6, 106.6)	106.2	(105.7, 106.7)	106.6	(106.1, 107.1)	105.1	(104.6, 105.6)	102.9	(102.4, 103.4)	101.7	(101.2, 102.2)
Saturated fat	28.3	(28.2, 28.5)	28.5	(28.4, 28.7)	28.4	(28.3, 28.5)	28.5	(28.4, 28.7)	28.6	(28.5, 28.7)	28.7	(28.6, 28.9)
Sodium	2.4	(2.34, 2.36)	2.4	(2.35, 2.38)	2.3	(2.26, 2.29)	2.3	(2.27, 2.30)	2.2	(2.23, 2.26)	2.3	(2.26, 2.30)
NSP Fibre	16.94	(16.88, 17.00)	16.81	(16.75, 16.88)	16.43	(16.37, 16.49)	16.64	(16.58, 16.70)	16.51	(16.44, 16.57)	16.62	(16.56, 16.68)
Carbohydrate excl. sugar	122.0	(121.5, 122.6)	119.5	(118.9, 120.0)	112.9	(112.4, 113.4)	112.6	(112.1, 113.1)	112.0	(111.4, 112.5)	113.3	(112.8, 113.8)

[1]Data are from 213,663,901 transactions reported by 50,672 households participating in Kantar Worldpanel UK between 2012 and 2017. Values are mean daily per capita energy and nutrient purchased to take-home. Some puddings, biscuits, and bread products, as well as all bacon and sausages, slimming products and milkshake mixes were excluded because of inconsistent nutrient information reported at product level. Products excluded could account for up 130kcal/capita/day each year.

Supplementary Table S2 - Changes in healthier food groups contributions to energy and nutrient purchased to take-home with 95% confidence intervals (Kantar Worldpanel UK 2012-2017)

			Δ 2017 - 2012[1]									
Healthier food groups[2]	Kcal 2012	(95% CI)	Energy (kcal)	(95% CI)	p-value	Fat (g)	(95% CI)	Protein (g)	(95% CI)	Sugar (g)	(95% CI)
Pasta, rice, potatoes and other grains	126.63	(125.77,127.49)	-18.12	(-19.32,-16.93)	<0.001	-0.18	(-0.20,-0.17)	-0.49	(-0.52,-0.45)	-0.17	(-0.18,-0.16)
Bread products	176.39	(175.39,177.39)	-13.33	(-14.67,-11.99)	<0.001	-0.13	(-0.15,-0.11)	-0.26	(-0.34,-0.18)	-0.15	(-0.17,-0.12)
Fruits	65.16	(64.78,65.53)	-1.06	(-1.60,-0.52)	<0.001	0.11	(0.10,0.12)	0.07	(0.06,0.07)	0.89	(0.77,1.00)
Milk	120.27	(119.22,121.32)	-0.18	(-1.74,1.38)	0.778	-0.32	(-0.40,-0.24)	0.24	(0.15,0.34)	0.30	(0.16,0.43)
Other dairy	19.95	(19.80,20.11)	-0.01	(-0.22,0.21)	0.797	0.02	(0.02,0.03)	0.06	(0.05,0.07)	-0.14	(-0.16,-0.11)
Legumes, nuts and seeds	26.62	(26.37,26.86)	0.30	(-0.08,0.68)	0.243	0.19	(0.16,0.22)	-0.10	(-0.12,-0.08)	-0.12	(-0.13,-0.10)
Sauces and condiments	2.44	(2.39,2.49)	1.13	(1.06,1.20)	<0.001	0.09	(0.08,0.09)	0.02	(0.02,0.03)	-0.02	(-0.02,-0.01)
Other drinks	8.36	(8.23,8.49)	2.10	(1.89,2.31)	<0.001	0.06	(0.05,0.06)	0.03	(0.02,0.05)	0.19	(0.15,0.22)
Ready meals and convenience food	107.48	(106.89,108.06)	2.56	(1.73,3.38)	<0.001	0.13	(0.09,0.16)	0.19	(0.16,0.22)	0.04	(0.02,0.05)
Proteins	75.56	(75.04,76.08)	3.89	(3.12,4.66)	<0.001	0.16	(0.11,0.20)	0.76	(0.66,0.87)	-0.03	(-0.04,-0.03)
Red meat	23.30	(23.10,23.50)	5.15	(4.87,5.42)	<0.001	0.24	(0.22,0.25)	0.47	(0.44,0.51)	0.00	(0.00,0.00)
Processed fish and meat	24.76	(24.56,24.96)	5.25	(4.94,5.57)	<0.001	0.21	(0.19,0.22)	0.40	(0.37,0.44)	0.02	(0.02,0.02)
Breakfast cereals	37.90	(37.63,38.17)	8.02	(7.59,8.44)	<0.001	0.07	(0.06,0.08)	0.19	(0.17,0.20)	0.21	(0.19,0.22)
Vegetables, excl. legumes and potatoes	42.03	(41.80,42.25)	9.97	(9.60,10.33)	<0.001	0.19	(0.17,0.21)	0.19	(0.18,0.21)	0.58	(0.54,0.61)

[1]Energy and nutrients changes given as /capita/day.

[2]Some puddings, biscuits, and bread products, as well as all bacon and sausages, slimming products and milkshake mixes were excluded because of inconsistent nutrient information reported at product level. Products excluded could account for up 130kcal/capita/day each year.

Supplementary Table S2 (continued) - Changes in healthier food groups contributions to energy and nutrient purchased to take-home with 95% confidence intervals (Kantar Worldpanel UK 2012-2017)

	Δ 2017- 2012[1]							
Healthier food groups[2]	Saturated fat (g)	(95% CI)	Sodium (g)	(95% CI)	NSP fibre (g)	(95% CI)	Carbohydrate excl. sugar (g)	(95% CI)
Pasta, rice, potatoes and other grains	-0.04	(-0.04,-0.04)	-0.01	(-0.01,-0.01)	-0.11	(-0.13,-0.09)	-3.35	(-3.59,-3.11)
Bread products	-0.02	(-0.02,-0.01)	-0.03	(-0.03,-0.03)	-0.26	(-0.29,-0.24)	-2.51	(-2.74,-2.28)
Fruits	0.05	(0.05,0.06)	0.00	(0.00,0.00)	0.06	(0.05,0.07)	-1.59	(-1.63,-1.55)
Milk	-0.12	(-0.17,-0.06)	0.01	(0.01,0.01)	-0.05	(-0.06,-0.05)	0.00	(-0.01,0.01)
Other dairy	0.01	(0.01,0.02)	0.00	(0.00,0.00)	-0.02	(-0.02,-0.02)	-0.07	(-0.08,-0.06)
Legumes, nuts and seeds	0.02	(0.02,0.03)	-0.01	(-0.01,-0.01)	-0.09	(-0.11,-0.08)	-0.33	(-0.37,-0.30)
Sauces and condiments	0.01	(0.01,0.01)	0.00	(0.00,0.00)	0.02	(0.02,0.02)	-0.01	(-0.02,-0.01)
Other drinks	0.02	(0.02,0.02)	0.01	(0.01,0.01)	-0.02	(-0.03,-0.01)	0.12	(0.11,0.13)
Ready meals and convenience food	0.07	(0.06,0.08)	0.00	(0.00,0.00)	-0.02	(-0.04,-0.01)	-0.22	(-0.34,-0.11)
Proteins	0.02	(0.01,0.03)	0.01	(0.01,0.01)	0.02	(0.01,0.02)	-0.04	(-0.06,-0.02)
Red meat	0.09	(0.09,0.10)	0.00	(0.00,0.00)	0.00	(0.00,0.00)	0.00	(0.00,0.00)
Processed fish and meat	0.04	(0.04,0.04)	0.01	(0.01,0.01)	0.00	(0.00,0.00)	0.20	(0.19,0.22)
Breakfast cereals	0.01	(0.01,0.01)	0.00	(0.00,0.00)	0.12	(0.11,0.14)	1.24	(1.17,1.31)
Vegetables, excl. legumes and potatoes	0.02	(0.02,0.03)	0.01	(0.01,0.01)	0.28	(0.26,0.29)	0.65	(0.62,0.69)

[1]Energy and nutrients changes given as /capita/day.

[2]Some puddings, biscuits, and bread products, as well as all bacon and sausages, slimming products and milkshake mixes were excluded because of inconsistent nutrient information reported at product level. Products excluded could account for up 130kcal/capita/day each year.

Supplementary Table S3 - Changes in less healthy food groups contributions to energy and nutrient purchased to take-home with 95% confidence intervals (Kantar Worldpanel UK 2012-2017)

			Δ 2017 - 2012[1]									
Less healthy food groups[2]	Kcal 2012	(95% CI)	Energy (kcal)	(95% CI)	p-value	Fat (g)	(95% CI)	Protein (g)	(95% CI)	Sugar (g)	(95% CI)
Fat and oil	162.87	(161.78,163.96)	-15.11	(-16.85,-13.38)	<0.001	-1.62	(-1.81,-1.43)	-0.03	(-0.03,-0.03)	-0.02	(-0.02,-0.02)
Caloric sweeteners	58.44	(57.90,58.99)	-11.37	(-12.15,-10.59)	<0.001	0.00	(0.00,0.00)	-0.01	(-0.01,-0.01)	-2.64	(-2.84,-2.45)
Breakfast cereals	44.80	(44.54,45.05)	-9.51	(-9.85,-9.18)	<0.001	-0.03	(-0.05,-0.02)	-0.19	(-0.20,-0.19)	-0.72	(-0.75,-0.69)
Red meat	24.34	(24.16,24.53)	-7.79	(-8.04,-7.54)	<0.001	-0.61	(-0.63,-0.59)	-0.67	(-0.69,-0.65)	-0.01	(-0.01,0.00)
Puddings and biscuits	184.12	(183.30,184.94)	-6.79	(-7.93,-5.65)	<0.001	-0.07	(-0.13,-0.02)	-0.18	(-0.19,-0.16)	-0.48	(-0.59,-0.37)
Juices	29.24	(29.00,29.48)	-6.12	(-6.45,-5.79)	<0.001	0.00	(0.00,0.00)	-0.06	(-0.06,-0.05)	-1.44	(-1.51,-1.36)
Ready meals and convenience food	57.89	(57.50,58.28)	-5.98	(-6.48,-5.48)	<0.001	-0.38	(-0.41,-0.34)	-0.10	(-0.12,-0.09)	-0.03	(-0.04,-0.02)
Other drinks	28.63	(28.33,28.93)	-4.79	(-5.24,-4.35)	<0.001	-0.01	(-0.02,0.00)	-0.04	(-0.05,-0.03)	-0.88	(-0.99,-0.78)
Alcohol	74.73	(73.81,75.64)	0.39	(-0.96,1.74)	0.682	0.03	(0.03,0.04)	0.00	(-0.01,0.01)	0.05	(-0.02,0.12)
Other morning goods	25.41	(25.17,25.66)	0.72	(0.39,1.04)	<0.001	0.00	(-0.02,0.02)	0.03	(0.02,0.04)	0.12	(0.10,0.14)
Other dairy	24.64	(24.39,24.89)	0.82	(0.45,1.18)	<0.001	0.17	(0.14,0.20)	-0.04	(-0.05,-0.03)	-0.16	(-0.19,-0.13)
Bread product	28.06	(27.87,28.25)	1.46	(1.19,1.73)	<0.001	0.01	(0.00,0.03)	0.02	(0.02,0.03)	0.03	(0.02,0.04)
Sauces and condiments	28.25	(28.03,28.47)	1.93	(1.58,2.28)	<0.001	0.23	(0.20,0.27)	0.02	(0.01,0.02)	-0.03	(-0.05,-0.01)
Chocolates and confectionary	105.25	(104.60,105.91)	3.83	(2.93,4.73)	<0.001	0.42	(0.37,0.47)	0.07	(0.05,0.08)	0.16	(0.04,0.27)
Processed fish and meat	36.53	(36.25,36.82)	3.95	(3.56,4.33)	<0.001	0.20	(0.17,0.23)	0.60	(0.57,0.63)	0.02	(0.02,0.02)
Cheese	59.20	(58.86,59.54)	5.95	(5.41,6.49)	<0.001	0.48	(0.44,0.53)	0.41	(0.37,0.44)	-0.08	(-0.08,-0.07)
Savoury snacks	75.57	(75.12,76.01)	7.41	(6.79,8.04)	<0.001	0.39	(0.35,0.43)	0.12	(0.10,0.13)	0.11	(0.10,0.12)
Table salt	-	-	-	-	-	-	-	-	-	-	-

[1]Energy and nutrients changes given as /capita/day.

[2]Some puddings, biscuits, and bread products, as well as all bacon and sausages, slimming products and milkshake mixes were excluded because of inconsistent nutrient information reported at product level. Products excluded could account for up 130kcal/capita/day each year.

Supplementary Table S3 (continued) - Changes in less healthy food groups contributions to energy and nutrient purchased to take-home with 95% confidence intervals (Kantar Worldpanel UK 2012-2017)

	Δ 2017- 2012[1]							
Healthier food groups[2]	Saturated fat (g)	(95% CI)	Sodium (g)	(95% CI)	NSP fibre (g)	(95% CI)	Carbohydrate excl. sugar (g)	(95% CI)
Fat and oil	-0.20	(-0.25,-0.16)	-0.03	(-0.03,-0.02)	-0.01	(-0.02,-0.01)	-0.04	(-0.05,-0.04)
Caloric sweeteners	0.00	(0.00,0.00)	0.00	(0.00,0.00)	0.00	(0.00,0.00)	-0.16	(-0.17,-0.15)
Breakfast cereals	-0.02	(-0.03,-0.02)	-0.02	(-0.02,-0.02)	-0.10	(-0.12,-0.09)	-1.41	(-1.46,-1.37)
Red meat	-0.31	(-0.32,-0.30)	-0.01	(-0.01,-0.01)	0.00	(0.00,0.00)	0.01	(0.01,0.01)
Puddings and biscuits	0.07	(0.04,0.10)	0.00	(0.00,0.00)	-0.08	(-0.09,-0.07)	-1.04	(-1.13,-0.95)
Juices	0.00	(0.00,0.00)	-0.01	(-0.01,-0.01)	-0.02	(-0.02,-0.01)	0.00	(-0.01,0.01)
Ready meals and convenience food	-0.13	(-0.14,-0.12)	-0.01	(-0.02,-0.01)	-0.08	(-0.09,-0.08)	-0.57	(-0.61,-0.53)
Other drinks	0.00	(-0.01,0.00)	-0.01	(-0.01,-0.01)	0.00	(0.00,0.01)	-0.26	(-0.29,-0.24)
Alcohol	0.02	(0.02,0.02)	0.00	(0.00,0.00)	0.00	(0.00,0.00)	0.40	(0.37,0.42)
Other morning goods	0.03	(0.02,0.04)	0.00	(0.00,0.00)	0.01	(0.00,0.01)	0.09	(0.05,0.13)
Other dairy	0.10	(0.08,0.12)	0.00	(0.00,0.00)	-0.01	(-0.01,-0.01)	0.00	(-0.03,0.02)
Bread product	0.02	(0.01,0.03)	0.00	(0.00,0.00)	0.01	(0.01,0.01)	0.16	(0.13,0.19)
Sauces and condiments	0.00	(0.00,0.01)	0.00	(0.00,0.01)	0.02	(0.02,0.02)	-0.05	(-0.07,-0.04)
Chocolates and confectionary	0.19	(0.16,0.21)	0.00	(0.00,0.00)	0.02	(0.01,0.02)	-0.46	(-0.49,-0.42)
Processed fish and meat	0.08	(0.07,0.10)	0.02	(0.02,0.02)	0.00	(0.00,0.00)	-0.11	(-0.12,-0.10)
Cheese	0.32	(0.29,0.35)	0.01	(0.01,0.01)	-0.02	(-0.02,-0.02)	0.08	(0.08,0.09)
Savoury snacks	0.01	(0.01,0.02)	0.01	(0.00,0.01)	0.06	(0.05,0.06)	0.58	(0.52,0.64)
Table salt	-	-	-0.03	(-0.04,-0.02)	-	-	-	-

[1] Energy and nutrients changes given as /capita/day.

[2] Some puddings, biscuits, and bread products, as well as all bacon and sausages, slimming products and milkshake mixes were excluded because of inconsistent nutrient information reported at product level. Products excluded could account for up 130kcal/capita/day each year.

Supplementary Figure S1: Data flow chart

Available sample: 50,672 active households reporting 225,032,065 product-level purchases of food and beverages between 2012 and 2017

↓

Correction of nutritional information (1.4%), measurement unit (0.09%) or pack number (0.1%)

Main products:

Eggs (1,399,417)	Crumpets and pikelets (146,629)
Baguettes (830,812)	Scones (30,682)
Naan breads (229,826)	Thins (27,705)
Pitta breads (179,052)	Buns (16,711)

Methods: 1) adjust reported nutritional information (per unit vs. per 100g); 2) use identical products at a different transaction; 3) use McCance and Widdowson's composition of food integrated dataset if unsure

↓

Exclusion of products with inconsistent nutritional information (5%):

5,667,244 desserts (e.g. mince pies, small tarts, cakes)
2,942,933 bacon and sausages
2,295,778 bread products (wholemeal light breads, ciabattas, bagels, crusty rolls, garlic breads, soft rolls, sausage rolls, stoneground wholemeal breads)
242,699 slimming products
124,814 muffins
94,696 milkshake mixes

↓

Analysis sample: 213,663,901 product-level purchases reported by 50,672 households
